# Library involvement in health informatics education for health professions students and practitioners: a scoping review

**DOI:** 10.5195/jmla.2021.1081

**Published:** 2021-07-01

**Authors:** Deborah L. Lauseng, Kristine M. Alpi, Brenda M. Linares, Elaine Sullo, Megan von Isenburg

**Affiliations:** 1 dlauseng@uic.edu, Regional Head Librarian & Assistant Professor, Library of the Health Sciences–Peoria, University of Illinois Chicago, Peoria, IL; 2 krisalpi@gmail.com, University Librarian, OHSU Library, Oregon Health & Science University, Portland, OR; 3 blinares@kumc.edu, School of Nursing Librarian, A. R. Dykes Library, University of Kansas Medical Center, Kansas City, KS; 4 elainej@gwu.edu, Associate Director, Reference and Instruction, Himmelfarb Health Sciences Library, George Washington University, Washington, DC; 5 megan.vonisenburg@duke.edu, Associate Dean, Library Services & Archives, Medical Center Library & Archives, Duke University Medical Center, Durham, NC

**Keywords:** health sciences libraries, educational services, librarian-educators, informatics education

## Abstract

**Objective:**

The purpose of this scoping review is to evaluate the extent of library or librarian involvement in informatics education in the health domain.

**Methods:**

We searched eight databases from their inception to 2019 for reports of informatics educational activities for health professionals or health professions students that involved library staff or resources. Two reviewers independently screened all titles/abstracts (n=2,196) and resolved inclusion decisions by consensus. From the full text of the 36 papers that met the inclusion criteria, we extracted data on 41 educational activities.

**Results:**

The most frequent coded purposes of activities were “teaching clinical tools” (n=19, 46.3%) and “technology” (n=17; 41.5%). Medical students were the most frequent primary audience (34.1%), though 41.5% of activities had multiple audiences. Evaluation was reported for 24 activities (58.5%), only a few of which assessed short or post-activity impact on attitudes, knowledge, or skills. The most common long-term outcome was applying skills in other courses or clinical experiences. Thematic analysis yielded three areas of outcomes and issues for the library and organizational partners: expanded opportunities, technology and resource issues, and value demonstration.

**Conclusions:**

Limited published examples of health informatics educational activities provide models for library roles in informatics education. More librarians should report on their informatics educational activities and provide sufficient details on the interventions and their evaluation. This would strengthen the evidence base about the potential impact of libraries within informatics education.

## INTRODUCTION

Medical informatics is considered an ambiguous domain within health sciences, and efforts to understand it in the context of educating health professionals are ongoing [1]. As technology evolved, health professions increased their focus on informatics skills for practitioners [2]. Librarians share an interest in advancing medical informatics and, in 1991, the Medical Library Association (MLA) started what is now known as its Medical Informatics Caucus [3]. The American Medical Informatics Association defines *health informatics* as composed of *clinical informatics* (including subfields such as medical, nursing, and dental informatics) and *public health informatics* [4]. We use medical informatics when that is the term used by the authors we are citing, but our review includes the broader domain of health informatics and all of its subfields.

The state of librarian involvement in medical informatics was documented in 2004 with King and MacDonald's 2002 survey of twenty-six informatics programs [5], which highlights librarians' roles in teaching within a successful medical informatics program. Evidence-based practice and information management were common, with personal digital assistants considered an interesting topic. The authors also discussed developing their informatics course, which expanded from typical library expertise areas to include telemedicine, decision analysis, and digital medical records. Librarians taught all these sessions, but no specific activities were reported.

Questions about involvement of librarians in health informatics remained salient in 2011 when the MLA Research Agenda authors [6] initiated a three-part Delphi study to identify the most important questions in health sciences librarianship, to then be addressed through literature reviews. Subsequently, volunteer teams formed to address the fifteen research questions identified [7], the eighth highest-ranked of which provided the basis for this review. Informatics was not clearly defined in the original question, as evident from a lower-ranked question that grouped informatics with more traditional library instructional domains: “What are the most effective instructional methods for teaching informatics/knowledge management/evidence-based practice in health sciences curricula?” Group 8 was assigned to address the two-part question, shown below, and our subgroup focused on the second part:

How do we provide information support in a clinical world that functions based on electronic medical records (EMR) systems and other similar informatics platforms and tools? *What is the library's role, if any, in providing preclinical education with respect to informatics applications like electronic medical records systems?* [6]

Librarians have published on partnerships with health professionals that led to effective educational programs for learning evidence-based medicine [8] and medical computing skills [9]. However, there are few examples of librarians practicing or teaching informatics beyond traditional information seeking and management, with one long-running example of librarian engagement in a nursing informatics program [10]. As part of the Integrated Advanced Information Management Systems (IAIMS) initiative, one library reported organizing pilot rotations for librarians in informatics to give them new career skills that can also be applied to educating health care providers [11]. Librarians also contributed to surveying the informatics skills of health professionals and students to understand opportunities for education [12] but did not explicitly call out a role for librarians in this training.

Following up on the King and McDonald study, King and Lapidus [13] published a subsequent survey intended to assess changes in the role of librarians in informatics education from 2004 to 2013. Librarians were included in non-library aspects of informatics training at 62% (34/55) of responding institutions. Topics classified as non-library included open web-based information, telemedicine and distance learning, clinical information systems, bioinformatics, decisions and decision-making, organizational informatics, public health informatics, mobile devices, emerging technologies, and introduction to research terminologies and ontologies. Furthermore, fifteen institutions reported librarians in leadership positions in their informatics programs. This institutional self-reported data presents one of the few attempts to document the role of librarians in medical informatics.

The purpose of this review is to address the question about the extent and nature of library involvement in health informatics education for current and future health professionals by describing the activities and outcomes that can be extracted from published papers. As this was a broad question for which we anticipated diverse types of literature, we chose to perform a scoping review [14].

## METHODS

We clarified the research question and expanded the definition of learners following preliminary database searches. The final question for which we based the search and inclusion/exclusion criteria was:

What is the role of librarians in providing education to clinicians/health practitioners and health professionals students in the use of informatics applications such as electronic health records (EHRs)/EMRs and clinical support tools (e.g., infobuttons, point of care tools)?

Because the question is open to a wide variety of articles reporting roles, we followed a scoping review methodology [14] rather than a more structured systematic review or replicating the survey research of King and Lapidus. The protocol was deposited with Open Science Framework [15]. Most of the reporting elements in the PRISMA Extension for Scoping Reviews (PRISMAScR) [16] are covered in the sections below. The search strategy developed with earlier members of the review group was peer-reviewed by the MLA Research Agenda leaders. The search included subject headings and keywords relevant to three primary concepts: librarians, informatics, and education (see Appendix A). Eight databases (CINAHL, ERIC, LISA, LISTA, PubMed, Scopus, Embase, and ProQuest Dissertations & Theses) were searched between March and April 2017, and searches of each database were updated November 15, 2019. No limits were applied. We reviewed abstracts for papers written in all languages, relying on our language skills or Google Translate to comprehend the work sufficiently to apply the inclusion/exclusion criteria.

### Inclusion/exclusion

The inclusion criteria addressed the three components of our research question. Included papers reported on an educational activity (e.g., curriculum, intervention, or materials created for learning purposes) that (1) had an audience of health-related practitioners or students in a health-related discipline; (2) contained content with some health informatics application, such as EHRs/EMRs or clinical support tools (e.g., infobuttons, point of care tools, e-prescribing); and (3) involved the participation of a librarian or library employee, which could include facilitating and hosting. Where the library includes non-library units, (e.g., the National Center for Biotechnology Information of the National Library of Medicine [NLM]), being employed by a library was not cause for inclusion. However, we allowed for non-librarian library employees such as technology specialists to be the activity provider.

The focus on health informatics led us to exclude bioinformatics, data science, evidence-based practice, literature searching, and general mobile applications as shown in the exclusion criteria below:

- No evidence that an educational activity took place (e.g., review or overview)
- Audience is primarily informatics or information professionals, not health practitioners or students
- Topic was only bioinformatics, data management or data science, literature/database searching, evidence-based practice, or general mobile apps
- No explicit library/librarian involvement in the educational activity
- No substantive description of the educational activity content (e.g., announcement/advertising of a course)

Papers often met more than one criteria; thus the PRISMA diagram presents the total number excluded rather than a by-criterion breakdown (see Appendix B for list of excluded papers).

We evaluated a test set to further refine the inclusion criteria. One author prepared a training set of fifty-two papers, two presumed to meet the inclusion criteria and fifty generated randomly through the use of Research Randomizer [17]. Using the criteria above, we indicated whether an article from this test set should be included in the full-text screening, excluded, or marked as unsure, which meant we would further discuss the abstract. Test agreement across all five reviewers was 46%. The twenty-nine (56%) marked “include” or “unsure” by at least one reviewer were discussed and the criteria refined. We discussed all citations marked “include” by any reviewer to come to consensus on whether they would advance to full-text review.

### Review

Titles and abstracts were independently reviewed by two novel assigned reviewers. Four authors (D.L.L., K.M.A., E.S., M.V.I.) completed the screening process with one author (B.M.L.) in reserve to resolve discrepancies. Each reviewer was paired at the same rate with each of the other reviewers. Full-text papers were independently reviewed by two reviewers different from those who screened the papers, and disagreements were resolved by consensus. We reviewed the bibliographies of all included papers, as well as overview articles not eligible for inclusion and did not identify additional articles for inclusion.

### Data extraction and analysis

For each included paper, two independent reviewers extracted available data on author affiliation, internal and external financial sponsorship, country in which the educational activity took place, the purpose of the educational activity, its audience(s), setting(s) and timing/frequency, study design or methods used, evaluation methods, learning outcomes, other outcomes, and next steps. Data available for extraction varied across the papers.

To enable quantitative characterization, we counted the extracted data as present or absent, assigned them to typologies (classifications based on types we created), or categorized them into themes that we developed from analyzing the extracted open text. A single author identified unique themes and developed typologies. Three authors (K.M.A., D.L.L., B.M.L.) then refined the definitions and agreed on the typologies to be applied. The extracted data were then independently coded by two authors, with the third author resolving differences. Evaluation methods were analyzed quantitatively as counts within an aggregated typology of what was being measured and how it was being measured and qualitatively through a thematic analysis of language from the text about why evaluation was or was not pursued and by whom.

The more extensive and diverse text extracted for outcomes and next steps was analyzed in a two-step process. One author (K.M.A.) derived a set of codes based on the extracted texts and then refined those codes into themes and grouped them into categories. This list of themes was provided to a second analyst (D.L.L.) to apply to the initial extractions and identify any content not covered for which additional codes would be needed. Codes only became themes if present in more than one paper. The two analysts negotiated the final list of themes through consensus. Details on assessing learner outcomes were converted to a checklist as part of the evaluation analysis. The final categories with exemplar quotes and their associated themes are presented in the results.

## RESULTS

**Figure 1:**
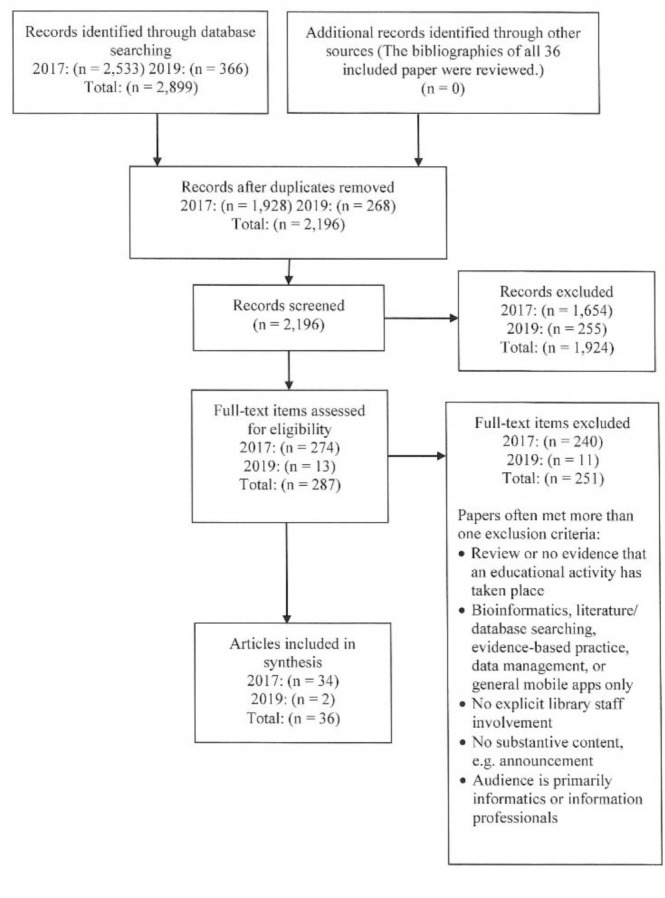
PRISMA Flowchart of literature search and study selection

The PRISMA flow diagram appears as Figure 1. The initial 2017 search results included 1,928 de-duplicated papers. After the first 500 papers were reviewed, discrepancies were discussed. Title/abstract screening agreement among reviewers was 87%. In title/abstract screening, eleven additional duplicates were identified. A total of 274 papers were selected for full-text screening. A total of 237 were excluded based on our criteria and three duplicates, resulting in thirty-seven eligible papers. During data extraction, three papers were eliminated for lacking the data elements to meet the inclusion criteria of reporting on an educational activity. This left thirty-four included papers. In 2019, an additional 268 unique results from the updated search were independently screened, resulting in thirteen papers eligible for full-text review, from which we included two papers. A total of thirty-six papers were identified for this scoping review.

### Librarian involvement in the production of the included papers

The thirty-six included papers addressed forty-one educational activities or interventions, hereafter referred to as “activities” (see Appendix C). Most were published for a librarian audience, the most common venues being *Journal of the Medical Library Association* (n=11, 30.6%) and *Medical Reference Services Quarterly* (n=10, 27.8%).

#### Authorship

The number of paper authors ranged from one to ten, with a mean of 3.2 (SD 2.4). A librarian author appeared to be the first author for twenty-nine (80.6%) papers. Librarian position titles were not consistently provided in the journal author affiliation field data, appearing in only twenty-four (66.7%) papers. Two librarians had informatics in their titles—one was a health informatics fellow and the other was an associate director who had an appointment as an assistant professor of medical informatics. While the most common titles were in the reference/education/information/instruction/liaison category, there were a similar number of director/associate director/assistant director/head positions, as well as one from information technology and one from cataloging. All but one of the reporting libraries were in the United States; the remaining library was in New Zealand.

#### Sponsorship

Nine (28.1%) papers noted funding from outside the parent institution. One mentioned the NLM Informatics Course, formerly NLM Biomedical Informatics Course at Woods Hole, as a starting point [5], and another stated that the three authors received Woods Hole fellowships [18]. Ten (27.8%) papers acknowledged internal institutional funding, primarily from collaborating partners. Financial contribution from the library was not included as sponsorship.

### Educational activities and interventions

Of the forty-one individually coded activities, two were reported using a case study design [19, 20] and one as an evaluative study [21]. The rest (n=38, 92.7%) were presented as program descriptions rather than having a study design; however, seven (19.5%) of these noted some use of assessment and evaluation strategies.

#### Purpose and audience

We were able to identify a purpose for all but one of the forty-one activities. Analysis of the extracted purpose data revealed five typologies in alignment with our inclusion criteria: “teaching clinical tools,” “technology,” “EHR/clinical decision support (CDS) use,” “resources and tool development,” and “informatics (not otherwise specified).” Teaching clinical tools involves teaching resources that are linked to the EHR (e.g., UpToDate, DynaMed), to CDS, or on handheld devices for clinical use, whereas EHR/CDS use focused on teaching the system itself. Technology includes knowledge of and skills in using hardware, software, or devices. Resources and tool development covers resources or tools developed for EHR/CDS/other clinical informatics platforms. Informatics captures general, unspecified informatics topics, or ethical/social issues/standards for information use in health care.

Half (n=20/40, 50.0%) of the activities had a single purpose, while the other half had two or more purposes. The primary purpose was teaching clinical tools (n=19, 47.5%), followed by technology (n=17; 42.5%). Further, teaching clinical tools was often paired with at least one other purpose (n=14/19), which was most frequently technology (n=12/14, 85.7%). Librarians taking a lead role in teaching personal digital assistants between 2001 and 2010 [22–27] represented just over a third (35.3%) of the technology purposes (n=6/17).

We anticipated a wide range of professional student and faculty audiences but found data reported for only ten unique audience groups. Medical students represented the primary audience for 14 activities (34.1%), followed by physicians (n=8, 19.5%) and nursing students (n=4, 9.8%). We noted a secondary audience in 17 activities (47.0%), most frequently residents/fellows (n=4/17, 23.5%). When including both primary and secondary audiences, eight activities included multidisciplinary audiences. Only nineteen activities provided numbers of participants.

Analysis of educational purpose by primary audience (Table 1) shows that four of the five purposes were offered to at least six different audiences. Informatics covered 80.0% of the audience groups, while teaching clinical tools and technology each covered 70.0%. EHR/CDS use covered just over half (60.0%). Resources and tool development was less common (30.0%) and often customized for a specific audience.

**Table 1:** Purpose of educational activities (n=40) according to their primary audience

Educational Purposes*/Primary Audience	Teaching Clinical Tools	Technology	EHR/Clinical Decision Support (CDS) Use	Resources & Tool Development	Informatics (not otherwise specified)	Total
Health Professional Students						
Medical	7	5	6	0	6	24
Nursing	3	2	1	0	1	7
Allied Health	0	0	0	0	1	1
Public Health	0	0	0	0	1	1
Other	1	1	1	0	1	4
Residents/Fellows	2	2	0	0	0	4
Health Professionals						
Physicians	3	4	2	4	1	14
Nurses	2	1	0	0	1	4
Clinical Informatics Team	0	0	1	1	0	2
Other	1	2	2	2	1	8
Total	19	17	13	7	13	69

* Half of the activities included more than one coded educational purpose

#### Setting and purpose

Details about the educational setting were included in nearly all of the activities (n=38). Fourteen occurred in a hospital or clinical setting; eighteen were held in an academic setting, of which ten also included an in-library setting; and six noted only a library setting. Twenty-nine (76.3%) of the activities were in-person activities, five (13.2%) involved a combination of in-person and virtual activities, and four (10.5%) were virtual only. In-person activities included the use of classrooms (n=8), conference rooms/offices (n=7), clinics (n=3), simulation labs (n=2), as well as unspecified locations (n=9).

Within the hospital/clinical setting, there was wider distribution of the five purposes, with teaching clinical tools being the highest and Informatics the lowest (Table 2). Resource and tool development activities only occurred with the hospital/clinical setting. Teaching clinical tools and informatics were the primary activities in the academic setting, while technology was the primary activity within the library setting. For activities occurring in combination between the library and an academic setting, the blending of in-person and virtual-asynchronous was seen in informatics, technology, and teaching clinical tools.

**Table 2:** Setting of educational activities (n=38) according to their purpose

Setting	In the library (only)			Academic setting (combined outside and inside the library)			Academic setting (only outside the library)			Hospital/clinical setting (outside of library)			
Purpose*	In-person	Virtual	Blended†	In-person	Virtual	Blended†	In-person	Virtual	Blended†	In-person	Virtual	Blended†	TOTALS
Informatics (not otherwise specified)	2	0	0	1	2	2	4	1	0	1	0	0	13
Technology	5	0	0	1	0	2	2	1	0	5	0	0	16
EHR/CDS Use	1	0	0	1	1	0	2	1	0	6	0	0	12
Teaching Clinical Tools	1	0	0	1	0	2	5	1	0	8	0	0	18
Resources & Tool Development	0	0	0	0	0	0	0	0	0	5	0	0	5
TOTALS	9	0	0	4	3	6	13	4	0	25	0	0	64

* Purpose – For five identified purposes coded within three activities, no setting was noted

† Blended is a combination of in-person and virtual-asynchronous

### Library involvement in collaborations

To understand library/librarian involvement, we examined the types and roles of all collaborators involved. We also categorized several types of non-library collaborators, from health sciences faculty, to hospital administrators, to information technology (IT) professionals. Over 90% of the thirty-six papers (n=33) involved at least one non-library collaborator, with nearly all listing multiple collaborators (n=29). The number of collaborators per activity ranged from one to seven, with a median of three. Health sciences faculty were the most frequently stated collaborators (n=26, 72.2%), followed by IT staff or technology experts (n=14, 38.9%) and health professionals without faculty roles, including those based at hospitals (n=12, 33.3%). Interprofessional collaboration was coded for just over one-third of the papers (n=13, 36.1%). Researchers or employees of state-level organizations were least frequently reported (n=2, 5.6%).

#### Roles of non-library collaborators

Of the thirty-three papers that mention non-library collaborators, over 60% (n=20) identified specific collaborator roles. Content contribution (n=14/20, 70.0%) was most often mentioned, followed by training/curriculum development (n=12/20, 60.0%). Other collaborator roles included trainer/presenter (n=7), coordinator (n=7), advocate or support provider (n=6), training/curriculum evaluation (n=6), and training/curriculum approval (n=5). Less common roles were research or grant proposal involvement (n=3), promotion or marketing (n=2), obtaining or approving continuing education (n=1), and user support (n=1). The number of stated collaborator roles ranged from none to six, with a median of three.

#### Library organizational or facility-based roles

Nearly 70% (n=25) of papers indicated one or more organizational or facilities-based roles for the library. The most frequent organizational roles for the library were curriculum/program planning and providing instructors/graders (n=9 each). The library also provided technology-related support, including providing technology, software or apps, or licensed or external e-resources and tools. Membership on committees and task forces from an organizational perspective was noted in 16.7% (n=6) of papers. Four papers (11.1%) reported hosting new web pages or LibGuides.

#### Library personnel roles

Nearly a third of the forty-one activities (n=13, 31.7%) reported a single library employee involved. Slightly over half (n=23, 56.1%) involved more than one library employee. Of those involving multiple library staff, eight activities indicated the specific number of employees, ranging from two to five. Release time for library employees was noted in five (13.9%) papers, while another eleven (30.6%) implied release time.

Most activities (n=34) detailed the library personnel roles. The most frequent roles were instructional content/assignment development (n=27), teach/train informatics (n=23), team teach/train (n=17), and leadership (e.g., faculty lead, coordinator; n=13). Nine activities included the role of vendor relations, and four noted the role of providing access to nontraditional online resources. The number of library personnel roles per activity ranged from one to six, with a median of three. The most frequently noted role, instructional content/assignment development, was often mentioned in the same paper along with teach/train informatics (n=23/27, 85.2%), team teach/train (n=17/27, 63.0%), and leadership (n=13/27, 48.2%).

### Educational interventions

To understand the extent of the intervention, we captured the activity structure. Most common were formal academic courses (n=9, 22.0%) and one-time presentations (n=8, 19.5%). Other structures included workshops (7.3%), self-directed and distance learning (7.3%), simulation training and clinical hours (7.3%), continuing education in either multiple or one-on-one sessions (4.9% each), and subject guides (4.9%).

#### Evaluation methods

Paper authors evaluated what attendees learned (n=11, 30.6%) less often than reporting how learners evaluated the activity (n=23, 63.9%), with nine evaluating both (25.0%). Evaluation strategies were described as part of an activity's needs assessment, and two articles described evaluation plans for future execution [27, 28]. Assignments, quizzes, and grades were typical ways of evaluating learners, with three reporting pre-/post-testing. Two examples of peer evaluation were offered. Continuing professional development credit was offered for only one [23] of the fourteen activities that involved health professionals. One institution was pursuing Magnet status [27].

Evaluations of activities commonly included surveys (n=16/23, 69.6%). Eight evaluations mentioning timing (22.2%) included two occurring over time, one mid-course, and one occurring one-month post-course. Response rates and results were infrequently reported (n=8, 22.2%). The nature of feedback was reported in four cases: three were positive/met expectations and one was mixed. Other evaluation approaches, mentioned once each, were participant satisfaction, future certification (NCLEX-RN) pass rates [27], the opinion of the course director, and comments from content retention exams. Examining usage data for electronic tools was mentioned in two papers [29]. Additionally, one paper measured the learners' use of and satisfaction with equipment [30]. A plan to combine four years of individual exam and activity evaluation data for a longitudinal evaluation of the impact of curriculum change was reported by one article as next steps [31].

### Outcomes for learners and the library/organization

Authors generally reported two types of outcomes: (1) learner achievement outcomes within and post-activity, and (2) library/organization outcomes and issues. Within learner achievement categories, specific themes were identified for changing attitudes, increasing knowledge, and developing skills. Table 3 lists the themes and gives an exemplar quote for each, including the post-activity observations. As content areas were already classified in the purpose typology, they were not thematically analyzed even though they often appeared together, such as in this quote:

The majority reported feeling greater competence and confidence in Medical Informatics as a result of the course, particularly with regard to the challenges of the electronic medical record (EMR), scholarly communication, information access issues, definition of MI, and description of issues of information storage and retrieval [32].

Table 4 represents themes within three categories of library/institutional outcomes and issues: expanded opportunities, technology and resource issues, and value demonstrations. Expanded opportunities were most common across papers and involved curriculum integration or course revision, expanding audiences, roles or collaborators, and addressing activity-generated follow-ups. Outcomes were generally presented positively, such as “The joint efforts strengthened team spirit and the relationship …” [30]. Individual costs toward achieving outcomes were rare, but an example from Ellero is illustrative, “The pain took the form of much self-initiated learning, deferring library projects, encountering scheduling conflicts, and expanding personal comfort zones.” [33]

## DISCUSSION

This scoping review relies on published papers from which we could extract intervention details to provide insight into informatics educational activities pursued by librarians independently and as collaborators. We assumed that librarian involvement in informatics education would steadily increase as new tools became available, the requirements for educational exposure to informatics expanded, and more librarians were trained in informatics through the NLM informatics course [9, 34]. While at least two papers cited NLM-funded training as an impetus for their involvement in informatics education, the low number of papers in this review does not suggest overall growth.

Comparing our examples with the informatics education literature broadly, the single institution case study or evaluation report of an educational intervention [35, 36] remains a primary type of published informatics education literature. Challenges with reporting depth and article focus have been observed in other informatics education literature, specifically He and colleagues who suggest that EHR training articles mainly report best practice principles or successes or failures while underreporting the process of training development [37].

### Limitations

The thirty-six papers we included represent a 1.6% inclusion rate from our search, a very low precision in order to maximize recall. Broad use of the term informatics to represent information science or other information-focused disciplines retrieved many irrelevant papers. Another challenge was that relevant papers did not report on a specific activity. One such example reported students developing evidence-based content intended for informatics tools, such as order sets, but stopped short of involving students with the informatics application [38]. Several papers on teaching data science [39] were retrieved in 2019 but excluded because we judged data science to be broader than the health informatics criteria. Although we did not hand-search any informatics journals, we do not think this would have yielded more studies than found by our searches and reference list checking.

The positive nature of the outcomes may reflect publication bias towards public reporting of only successful collaborations. The picture of what has been done might be expanded if meeting presentations or grey literature were included. However, we wanted to focus on what informatics educators would find about working with library partners, and MLA presentations are not generally accessible to nonmember audiences.

### Comparisons with prior surveys of library educational activities

We compared our data on types of informatics training and roles of librarians with findings reported by King and Lapidus [13]. Their question on the delivery of courses resulted in varied responses among their 32 responding institutions: 71.8% provided classroom instruction only, 12.5% online only, <1.0% a hybrid combination of classroom and online, and 12.5% offered multiple courses that included a variety of methods. We found roughly comparable proportions of different settings across the 41 educational activities covered in this review. Like King and Lapidus, classrooms/conference rooms served as the primary setting (29.2%) both outside and inside the library. Unlike King and Lapidus, however, higher proportions of educational settings covered in this review had virtual (24.3%) or blended/hybrid settings (9.8%) settings. Possible explanations include librarians embracing new instructional methodologies or changes in educational demands of health professionals and students.

In examining librarian roles, the King and Lapidus survey asked the thirty-four institutions with librarian involvement in non-library aspects of informatics instruction to identify the roles played by librarians. For teaching, they reported library-centered programs taught exclusively by librarians (41%) separately from guest speaking by librarians in classes in other departments (82%). In comparison, we found librarian roles of instructional involvement connected with instructional content/assignment development (65.9%), teaching/training informatics (56.1%), and team teaching/training (41.5%). Almost half (44%) of King and Lapidus' respondents identified librarians in a leadership or coordination role for an interdisciplinary team for at least one course—these roles included “leading a second-year medical student evidence-based medicine team; being the course director of an interdisciplinary evidence-based medicine course; chairing a medical informatics and bioinformatics committee; coordinating a seminar series on ethics for interdisciplinary students; and being the director of a graduate-level certificate program” [13]. Similarly, we identified leadership roles in 38.2% of activities. King and Lapidus also asked about library support for informatics courses taught by other faculty (26%), which may be comparable to our instructional content/assignment development category or be a collective grouping of other roles we identified such as assessment development, vendor relations, tech support, access to online resources (beyond traditional library resources), tool development (informatics), and consultations (non-tech). This variety represents a branching out of library engagement with informatics education beyond instruction. One final role evident from our review that was not discussed by King and Lapidus was authorship of an informatics educational activity paper; 80.6% of our papers included a librarian coauthor.

While the Nevius et al. survey on educational activities by health sciences libraries does not include informatics as a category, training on apps, which we considered an informatics educational activity, was the most common write-in response [40]. This corresponds with handheld computing as the focus of six of our included studies published between 2001 and 2010. Librarians' successful educational contributions to handheld computing may have led to further collaborations on training the future generation of these tools, but if it has, it seems to have moved out of informatics education and into the evidence-based practice domain.

### Implications for intervention reporting

Complete reporting of intervention details in educational or instructional activities is essential to enable educators to translate research evidence into practice. Most papers were program descriptions and not designed as research studies of educational interventions. There were very few mentions of study design—only two case studies and some pre- and post-surveys for evaluation purposes. Additionally, several papers reported on multiple complementary activities. While this realistically represented the continuum of librarian involvement, combining the overall impact of these multiple activities often made it unclear how each contributed or was evaluated.

The data elements we chose to extract from the included studies were similar to many of those TIDieR (template for intervention description and replication) checklist [41]. Albarqouni, Glasziou, and Hoffman used the TIDieR checklist of ten to twelve elements to assess the completeness of intervention reporting before March 2016 and found that none of the educational studies in evidence-based practice they reviewed completely reported all of the main items of the educational intervention [42]. Although our study did not evaluate the use of TIDieR items, the majority of our included studies addressed these ten TIDieR elements in different terms: why; procedures; who provided; how; where; when and how much; tailoring; modifications; how well: planned; and how well: actual. TIDieR elements not commonly present were a name or phrase that described the intervention, clear demarcation between multiple interventions, and information on accessing the intervention educational materials.

Instructions to authors and publication types of the journals at the time of submission may have influenced the reporting depth. Several of the papers in *Medical Reference Services Quarterly* were published as part of a column which launched in 1994 as “Medical Informatics Education,” later separated into two separate columns on “Medical Informatics” and “Education and Training,” and merged again in 1999 as “Informatics Education” [43]. In 2020, wide variability remains from requirements to use the Medical Education Research Study Quality Instrument (MERSQI) to structured reports of less than 500 words in *Medical Education's* twice-yearly publication “Really Good Stuff: Lessons learned through innovation in medical education” [44]. The three elements covered in these reports, (1) what problem was addressed; (2) what was tried; and (3) what lessons were learned, represent the essentials for libraries reporting on informatics educational activities in future publications.

### Conclusions

This scoping review evaluated the extent of library or librarian involvement in informatics education for health practitioners and health professional students. There are limited published examples of health informatics educational activities that libraries can use as models for librarian roles in informatics education for health professionals and students. Those we reviewed are predominantly encouraging in terms of the successful outcomes and demonstrations of value added by the library.

The paucity of included studies and activities demonstrates the need for more libraries to report on these educational activities, with sufficient details on the interventions and evaluation. We encourage library staff contributing to informatics education for health professions students and practitioners to report their efforts in the published literature, further strengthening the evidence base about the potential impact of libraries within informatics education.

## Data Availability

The spreadsheet with the coded data from the 36 included papers for this project is publicly available within the University of Illinois Chicago INDIGO repository, https://indigo.uic.edu/, DOI: http://dx.doi.org/10.25417/uic.14622099. The spreadsheet of extended quotes of extracted papers data from the included papers represent a substantial proportion of the copyrighted and therefore cannot be made publicly available. This extracted data will be made available to researchers upon request to the first author.

## SUPPLEMENTAL FILES

**Table 3** Short-term- and post-activity outcomes with exemplar quotes

**Table 4** Library and organizational-level issues and outcomes represented by themes with exemplar quotes

**Appendix A** Search strategies

**Appendix B** Excluded papers

**Appendix C** Included papers
