# Supplementary material for: Library involvement in health informatics education for health professions students and practitioners: a scoping review
Source: J Med Libr Assoc. 2021 Jul 1;109(3):365–75. doi: 10.5195/jmla.2021.1081 (PMC8485947; doi:10.5195/jmla.2021.1081)
Supplement: Supplementary file 1 — Table 3 Short-term- and post-activity outcomes with exemplar quotes [file jmla-109-3-365-s01.docx]

**Table 3** Short-term and post-activity outcomes with exemplar quotes

| Outcomes  (Aspirational or Achieved) | Exemplar quote (author name, publication year, page) |
| --- | --- |
| **Short-Term Activity Outcomes** | |
| ATTITUDES | |
| Confidence | Typically, those who define themselves as marginally computer literate become more confident and interested; those who are already expert computer users learn new, specifically medical, applications. (Hannigan et al., 1996, p. 85) |
| Interest | Anecdotally, many students were pleasantly surprised by the course content which sparked new interest in topics such as health literacy and scholarly communication. (Burnette, DeGroote & Dorsch, 2012, p.63) |
| Perspective | The variety of perspectives brought to the table by diverse faculty members enriched the learning experience for students by revealing, in practice, informatics as a product of numerous disciplines. In addition, students were provided a living example of an essential health care skill, working within a multidisciplinary team. (King, Murray & MacDonald, 2010, p. 111) |
| KNOWLEDGE | |
| Awareness/understanding of applicability | The students have an additional opportunity to increase their informatics knowledge… (Harrod & Gomes, 2017, p. 118) |
| Learned through networking | The small groups allowed participants to engage, collaborate, and network with each other; discuss the challenges of bioinformatics; and address issues they face in their everyday work environment. (Carlson, 2017, p. 255) |
| Preparedness to apply knowledge | Paired t-tests showed highly significant increases in the overall preparedness levels for physicians and nurses (p < 0.05). Therefore, the overall self-efficacy of physicians and nurses to use EMRs increased after simulation training as compared to before-simulation training. (Vuk et al., 2015, p. 426) |
| SKILLS | |
| Access new resources | Clinicians can easily search multiple resources with one click, thus broadening their information horizons by introducing them to new information resources that may provide better or more direct answers than their familiar information standbys. (Epstein et al., 2010, p. 226) |
| Discuss topics | There are lively conversations about citations, debates about the validity of data from clinical trials in various phases, and comparisons of drug interactions and laboratory values. (Schwartz, 2011, p. 80) |
| Install and/or use programs | Over the course of the training that focused on clinically experienced but techno-phobic attending physicians, library trainers worked with approximately 100 medical residents and students, loaded software application programs on their PDAs, and taught them how to use the programs. (Wallace, 2007, p. 35) |
| **Post-Activity Outcomes** | |
| Applied in other courses/clinical experiences | Results showed that students found them useful during their clerkships, preferred software that was easy to use and free, needed help with downloading and installing programs, and were unaware of how to protect data on their PDAs. (Crowell & Shaw-Kokot, 2003, p. 6) |
| Developed competency | Integrating knowledge-based medical information-searching skills where they naturally fit in the curriculum provides students with the opportunity to apply these skills in other courses and clinical experiences thereby enabling competency development. (Geyer & Irish, 2008, p. 460) |
| Promoted to others | Some members of the group have become almost evangelical about encouraging other students and faculty to use computer resources. (Hannigan & Edwards, 1996, p. 74-5) |
| Saved time | “…residents report spending significantly less time collecting patient information as well as having greater ability to access desired test results and orders at the point of care.” (Tomasko et al., 2014, p. 34) |
| Self-directed one’s lifelong learning | For students, use of handhelds provides one of the best implementations of the Association of American Medical College’s recommendation to foster self-directed, life-long learning, and developing information management skills. (Stoddard, 2001, p. 81) |
