# Supplementary material for: Library involvement in health informatics education for health professions students and practitioners: a scoping review
Source: J Med Libr Assoc. 2021 Jul 1;109(3):365–75. doi: 10.5195/jmla.2021.1081 (PMC8485947; doi:10.5195/jmla.2021.1081)
Supplement: Supplementary file 2 — Table 4 Library and organizational-level issues and outcomes represented by themes with exemplar quotes [file jmla-109-3-365-s02.docx]

**Table 4** Library and organizational-level issues and outcomes represented by themes with exemplar quotes

| Theme | Exemplar quote(s) (author name, publication year, page number) |
| --- | --- |
| **EXPANDED OPPORTUNITIES** | |
| Collaboration between library and other disciplines | The personal connections made between librarians and clinicians can lead to other future interactions. (Ellero, 2009, p. 101) |
| Course/activity content revisions or audience number or type expansions | The prerequisite for the course was changed from fourth-year standing to completion of the second year. (Burnette, DeGroote & Dorsch, 2012, p. 62) |
| Curriculum–add/change/sustain | “…the course director was able to keep the clinical FOSCE curriculum as planned while accommodating facility/time constraints, and the librarians had an invitation to both develop and teach new curricula.” (Harrod & Gomes, 2017, p. 118) |
| Increased communication and needs awareness | The process increased communication between surgeons and the Library and increased librarians’ awareness of the information needs and work practices of surgeons. (Tomasko et al., 2014, p. 34) |
| Librarian role expanded/more integrated | Based on the initial successful collaboration, the School of Medicine asked HSL staff to  help provide training sessions and updated information on resources for third- and fourth-year medical students. (Crowell & Shaw-Kokot, 2003, pp. 4-5) |
| New partner | Clinical agencies within the geographic area, and medical schools, have been excited to learn about the success of the group and have requested collaboration. (Griffin-Soebel et al., 2010, p. 43) |
| **TECHNOLOGY ISSUES/ DEVELOPMENT** | |
| Acquisition of resource, software or hardware | Student enthusiasm for SAM-CD and Iliad resulted in the purchase of those programs for the College’s Learning Resource Unit. (Hannigan & Edwards, 1996, p. 74) |
| Creation or modification of resource or interface | “…collaborations between a Department of Surgery, a Department of Information Technology, and an academic health sciences library resulted in the development of an electronic surgical library available at the bedside, the deployment of tablet devices for surgery residents, and implementation of a tablet-friendly user interface for the institution’s electronic medical record.” (Tomasko et al., 2014, p. 32) |
| Participation by clinicians in informatics learning | [Negative] “…staff encountered some difficulty recruiting physicians to participate....Often a person feels like he or she is the only one who lacks a particular skill, such as using a PDA, and spends considerable energy hiding this shortcoming from others, thereby making recruitment more difficult.” (Wallace, 2007, p. 35)  [Positive] All adjunct and clinical faculty have had time to learn and experience teaching with high-fidelity simulation, with positive feedback. (Griffin-Soebel et al., 2010, p. 43) |
| System access impact | [Negative]: The original plan to have students work with fictitious patient cases in TouchWorks in real time evolved into screenshots of TouchWorks in PowerPoint due to time and access limitations. (Gomes, Linton & Abate, 2013, p. 9)  [Positive]: The librarians' view of the EMR is identical to that of the physicians so that librarians can understand and assist physicians with their questions. (Welton, 2010, p. 219) |
| **VALUE DEMONSTRATIONS** | |
| Increased resource use | “…demonstrates increased usage following the marketing campaign and training.” (Knehans & Schirm, 2015, p. 157) |
| Increased service or consultation requests | “…what grew out of "Camp PDA" were individual consultations that are now done on a regular basis with not only students but also with residents and other clinical staff.” (Modica & Cuddy, 2008, p.186) |
| Marketing/promotion | [External] Already we have seen interest from other parties and schools to create a graduate level health informatics curriculum…As a result of participating in this course, we have been able to promote the VCU libraries’ teaching mission and resources beyond our immediate campus. (Turman, Self & Calarco, 2004, p. 24-5)  [Internal] Working with team members throughout the organization provided the best marketing for the library and the librarian (Miles, 2015, p. 237) |
| Relationship-building | The joint efforts strengthened team spirit and the relationship between the Library and the Department of Computer Medicine (Collins et al., 1992, p. 7) |
| Visibility and recognition | [External] Faculty from other schools, having heard of the project, have visited Georgetown and have requested the software program.  [Internal] Through this program, the librarians are receiving additional visibility and recognition from the faculty for their expertise in information management and technical teaching skills.  (both from Broering, 1991, p. 281) |
