# Supplementary material for: Library involvement in health informatics education for health professions students and practitioners: a scoping review
Source: J Med Libr Assoc. 2021 Jul 1;109(3):365–75. doi: 10.5195/jmla.2021.1081 (PMC8485947; doi:10.5195/jmla.2021.1081)
Supplement: Supplementary file 3 — Appendix A Search strategies [file jmla-109-3-365-s03.docx]

**Appendix A: Search strategies**

Eight databases were searched between March and April 2017.

Searches of each databases were updated November 15, 2019.

**CINAHL** (EBSCO; 1937-November 15, 2019)

**(**(MH "Informatics") OR (MH "Health Informatics+") OR (MH "Clinical Data Repository") OR (MH "Data Warehouse") OR (MH "Health Information Systems+") OR (MH "Computerized Patient Record") OR (MH "Electronic Order Entry") OR TI ( "information button" OR "information buttons" OR “infobutton” OR “infobuttons” OR “info button” OR “info buttons” OR EHR OR EHRs OR EMR OR EMRs OR "electronic health record" OR "electronic health records" OR "clinical decision support system" OR "clinical decision support systems" OR "point of care system" OR "point of care systems" OR ("point of care" AND technology) OR "bedside technology" OR "bedside computing" OR epic OR epiccare OR cerner OR allscripts OR nextgen OR centricity OR curemd OR "eClinical Works" OR "GE Healthcare" OR "Practice Fusion" OR "athena health" OR kareo OR "medical order entry" OR "computerized provider order entry" OR "computerized physician order entry" OR CPOE OR "electronic prescribing" ) OR AB ( "information button" OR "information buttons" OR “infobutton” OR “infobuttons” OR “info button” OR “info buttons” OR EHR OR EHRs OR EMR OR EMRs OR "electronic health record" OR "electronic health records" OR "clinical decision support system" OR "clinical decision support systems" OR "point of care system" OR "point of care systems" OR ("point of care" AND technology) OR "bedside technology" OR "bedside computing" OR epic OR epiccare OR cerner OR allscripts OR nextgen OR centricity OR curemd OR "eClinical Works" OR "GE Healthcare" OR "Practice Fusion" OR "athena health" OR kareo OR "medical order entry" OR "computerized provider order entry" OR "computerized physician order entry" OR CPOE OR "electronic prescribing" )**)** **AND** **(**(MH "Librarians+") OR (MH "Libraries+") OR AF (library) OR TI ( librarians OR librarian OR informationist OR informationists OR "information specialist" OR "information specialists" OR library OR libraries ) OR AB ( librarians OR librarian OR informationist OR informationists OR "information specialist" OR "information specialists" OR library OR libraries)**)** **AND** **(**(MH "Education+") OR TI ( educate OR education* OR educating OR teach OR teaching OR train OR training OR instruct OR instruction* OR instructing OR curriculum OR curricula OR workshop OR workshops) OR AB ( educate OR education* OR educating OR teach OR teaching OR train OR training OR instruct OR instruction* OR instructing OR curriculum OR curricula OR workshop OR workshops)**)**

**ERIC** (EBSCO; 1960-November 15, 2019)

(informatics OR "information button" OR "information buttons" OR infobutton OR infobuttons OR ehr OR ehrs OR emr OR emrs OR "electronic health record" OR "electronic health records" OR "electronic medical record" OR "electronic medical records" OR "clinical decision support system*" OR ("point of care” **AND** (technology OR system*)) OR epic OR epiccare OR cerner OR allscripts OR nextgen OR centricity OR "medical order entry system*" OR cpoe OR "electronic prescribing") AND (librarian OR librarians OR informationist OR informationists OR "information specialist" OR “information specialists” OR library OR libraries) **AND** (educate OR education OR educating OR teach OR teaching OR teacher OR train OR trainer OR training OR instruction OR instruct OR instructing)

**LISA** (ProQuest; 1969-November 15, 2019)

(SU.EXACT("Health informatics") OR SU.EXACT("Informatics") OR SU.EXACT("Electronic health records") OR AB,TI(informatics OR "information button" OR "information buttons" OR err OR errs OR emir OR emirs OR "electronic health record" OR "electronic health records" OR "electronic medical record" OR "electronic medical records" OR "clinical decision support systems" OR "clinical decision support system" OR "point of care systems" OR "point of care system" OR unbutton OR ("point of care" AND technology) OR "bedside technology" OR "bedside computing" OR epic OR epicarp OR cerner OR shellscripts OR roentgen OR centricity OR cured OR "clinical Works" OR "gey Healthcare" OR "Practice Fusion" OR "athena health" OR karoo OR "medical order entry systems" OR "medical order entry system" OR coe OR "computerized provider order entry" OR "computerized physician order entry" OR "electronic prescribing")) **AND** (SU.EXACT("Librarians") OR SU.EXACT("Embedded librarianship") OR SU.EXACT("Libraries") OR AB,TI(informations OR informations OR "information specialist" OR "information specialists" OR library OR libraries)) **AND** (SU.EXACT("Education") OR SU.EXACT("Training") OR SU.EXACT("User training") OR SU.EXACT("Instructional design") OR SU.EXACT("Teaching") OR SU.EXACT("Curriculum development") OR AB,TI(educate OR education OR educating OR teach OR teaching OR train OR training OR instruction OR instruct OR instructing OR curriculum OR curricula OR workshop*))

**LISTA** (EBSCO; 1960-November 15, 2019)

(MH "Informatics") OR (MH "Health Informatics+") OR (MH "Clinical Data Repository") OR (MH "Data Warehouse") OR (MH "Health Information Systems+") OR (MH "Computerized Patient Record") OR (MH "Electronic Order Entry") OR TI ( "information button" OR "information buttons" OR “infobutton” OR “infobuttons” OR “info button” OR “info buttons” OR EHR OR EHRs OR EMR OR EMRs OR "electronic health record" OR "electronic health records" OR "clinical decision support system" OR "clinical decision support systems" OR "point of care system" OR "point of care systems" OR ("point of care" AND technology) OR "bedside technology" OR "bedside computing" OR epic OR epiccare OR cerner OR allscripts OR nextgen OR centricity OR curemd OR "eClinical Works" OR "GE Healthcare" OR "Practice Fusion" OR "athena health" OR kareo OR "medical order entry" OR "computerized provider order entry" OR "computerized physician order entry" OR CPOE OR "electronic prescribing" ) OR AB ( "information button" OR "information buttons" OR “infobutton” OR “infobuttons” OR “info button” OR “info buttons” OR EHR OR EHRs OR EMR OR EMRs OR "electronic health record" OR "electronic health records" OR "clinical decision support system" OR "clinical decision support systems" OR "point of care system" OR "point of care systems" OR ("point of care" AND technology) OR "bedside technology" OR "bedside computing" OR epic OR epiccare OR cerner OR allscripts OR nextgen OR centricity OR curemd OR "eClinical Works" OR "GE Healthcare" OR "Practice Fusion" OR "athena health" OR kareo OR "medical order entry" OR "computerized provider order entry" OR "computerized physician order entry" OR CPOE OR "electronic prescribing" ) **AND** (MH "Librarians+") OR (MH "Libraries+") OR AF (library) OR TI ( librarians OR librarian OR informationist OR informationists OR "information specialist" OR "information specialists" OR library OR libraries ) OR AB ( librarians OR librarian OR informationist OR informationists OR "information specialist" OR "information specialists" OR library OR libraries ) **AND** (MH "Education+") OR TI ( educate OR education* OR educating OR teach OR teaching OR train OR training OR instruct OR instruction* OR instructing OR curriculum OR curricula OR workshop OR workshops) OR AB ( educate OR education* OR educating OR teach OR teaching OR train OR training OR instruct OR instruction* OR instructing OR curriculum OR curricula OR workshop OR workshops)

**PubMed** (NLM; 1946-November 15, 2019)

((((((((((informatics[Mesh:noexp] OR informatics[tiab])) OR ("information button"[all fields] OR "information buttons"[all fields] OR EHR[tiab] OR EHRs[tiab] OR EMR[tiab] OR EMRs[tiab] OR "Electronic Health Records"[Mesh:noexp] OR "electronic health record"[all fields] OR "electronic health records"[all fields])) OR ("Decision Support Systems, Clinical"[Mesh:noexp] OR "clinical decision support system"[all fields] OR "clinical decision support systems"[all fields])) OR "Point-of-Care Systems"[Mesh:noexp]) OR ("point of care system"[all fields] OR "point of care systems"[all fields] OR ("point of care"[all fields] AND technology[all fields]))) OR ("bedside technology"[all fields] OR "bedside computing"[all fields]) OR (epic[tiab] OR epiccare[tiab] OR cerner[tiab] OR allscripts[tiab] OR nextgen[tiab] OR centricity[tiab])) OR ("Medical Order Entry Systems"[Mesh:noexp] OR "medical order entry systems"[all fields] OR "computerized provider order entry"[all fields] OR "computerized physician order entry"[all fields] OR CPOE[tiab] OR "Electronic Prescribing"[Mesh:noexp] OR "electronic prescribing"[all fields]))) **AND** (Librarians[Mesh:noexp] OR librarians[all fields] OR librarian[tiab] OR informationist[tiab] OR informationists[tiab] OR "information specialist"[tiab] OR "information specialists"[all fields] OR library[tiab] OR library[ad] OR Libraries[Mesh] OR libraries[all fields])) **AND** (Education[Mesh:noexp] OR educate[all fields] OR education[all fields] OR educating[all fields] OR Teaching[Mesh:noexp] OR teach[all fields] OR teaching[all fields] OR train[all fields] OR training[all fields] OR instruct[all fields] OR instruction[all fields] OR instructing[all fields] OR curricula[all fields] OR curriculum[all fields])

**Scopus** (Elsevier; 1970-November 15, 2019)

( TITLE-ABS-KEY ( informatics OR "information button" OR "information buttons" OR ehr OR ehrs OR emr OR emrs OR "electronic health record" OR "electronic health records" OR "electronic medical record" OR "electronic medical records" OR "clinical decision support systems" OR "clinical decision support system" OR "point of care systems" OR "point of care system" OR infobutton OR ("point of care" AND technology) OR "bedside technology" OR "bedside computing" OR epic OR epiccare OR cerner OR allscripts OR nextgen OR centricity OR curemd OR “eClinical Works” OR “GE Healthcare” OR “Practice Fusion” OR “athena health” OR Kareo OR "medical order entry systems" OR "medical order entry system" OR cpoe OR "computerized provider order entry" OR "computerized physician order entry" OR "electronic prescribing" ) ) **AND** ( TITLE-ABS-KEY ( librarian OR librarians OR informationist OR informationists OR "information specialist" OR "information specialists" OR library OR libraries ) ) **AND** ( TITLE-ABS-KEY ( educate OR education OR educating OR teach OR teaching OR train OR training OR instruction OR instruct OR instructing OR curriculum OR curricula OR workshop* ) )

**Embase** (Elsevier; 1974-November 2019)

((informatics OR 'information button' OR 'information buttons' OR ehr:ab,ti OR ehrs:ab,ti OR emr:ab,ti OR 'electronic health record'/de OR 'electronic health record' OR 'electronic health records' OR 'electronic medical record' OR 'electronic medical records' OR 'electronic medical record system'/de OR 'computerized medical records systems':ab,ti OR 'clinical decision support system'/de OR 'clinical decision support system':ab,ti OR 'clinical decision support systems' OR 'point of care systems'/de OR infobutton OR infobuttons OR 'point of care system' OR 'point of care systems' OR ('point of care' AND technology) OR 'bedside technology' OR 'bedside computing' OR epic:ab,ti OR epiccare:ab,ti OR cerner:ab,ti OR allscripts:ab,ti OR nextgen:ab,ti OR centricity:ab,ti OR curemd:ab,ti OR 'eclinical works':ab,ti OR 'ge healthcare':ab,ti OR 'practice fusion':ab,ti OR 'athena health':ab,ti OR kareo:ab,ti OR 'physician order entry system'/de OR 'physician order entry system' OR 'medical order entry system' OR

'computerized provider order entry'/de OR 'computerized physician order entry' OR cpoe:ab,ti OR 'electronic prescribing'/de OR 'electronic prescribing') **AND** ('librarian'/de OR librarian:ab,ti OR librarians OR informationist:ab,ti OR informationists:ab,ti OR 'informational specialist':ab,ti OR 'information specialists' OR 'library'/de OR library:ab,ti OR library:ad OR libraries) **AND** ('education'/de OR educate OR education* OR educating OR 'teaching'/de OR teach OR teaching OR train OR training OR instruct OR instruction* OR instructing OR curricula OR curriculum OR workshop*) ) NOT [medline]/lim

**ProQuest Dissertations & Theses** (ProQuest; 1861-November 15, 2019)

(informatics OR information button OR information buttons OR ehr OR ehrs OR emr OR emrs

OR electronic health record OR electronic health records OR electronic medical record OR electronic medical records OR clinical decision support system* OR (point of care AND (technology OR system*)) OR epic OR epiccare OR cerner OR allscripts OR nextgen OR centricity OR medical order entry system* OR cpoe OR electronic prescribing) **AND** (librarian* OR informationist* OR information specialist OR library OR libraries) **AND** (educate OR education OR educating OR teach OR teaching OR teacher OR train OR trainer OR training OR instruction OR instruct OR instructing)
