# Supplementary material for: Library involvement in health informatics education for health professions students and practitioners: a scoping review
Source: J Med Libr Assoc. 2021 Jul 1;109(3):365–75. doi: 10.5195/jmla.2021.1081 (PMC8485947; doi:10.5195/jmla.2021.1081)
Supplement: Supplementary file 5 — Appendix C Included papers [file jmla-109-3-365-s05.docx]

**Appendix C:** Included papers

| **Number of Activities Extracted**  (n=41) | **Included Papers**  (n=36) |
| --- | --- |
| 1 | Airth-Kindree N, Vandenbark RT. Mobile applications in nursing education and practice. Nurse Educ 2014;39(4):166-9. |
| 1 | Broering NC. The MAClinical Workstation Project at Georgetown University. Bull Med Libr Assoc 1991;79(3):276-81. |
| 1 | Burnette MH, De Groote SL, Dorsch JL. Medical informatics in the curriculum: development and delivery of an online elective. J Med Libr Assoc 2012;100(1):61-3. |
| 1 | Carlson K. Librarian participation in biomedical informatics education and practice. J Hosp Librariansh 2017;17(3):254-9. |
| 1 | Collins B, Linton A, Merril J, Pomerantz K, Winthrop S. Introducing computer literacy skills for physicians. Med Ref Serv Q 1992;11(4):1-9. |
| 3 | Crowell K. Shaw-Kokot J. Extending the hand of knowledge: promoting mobile technologies. Med Ref Serv Q. 2003;22(1):1-9. |
| 1 | Cyrus J, Duggar DC, Esparza J, Adams M, Dobbins M, Pullen K. Connecting with hospital nurses through MINE. J Hosp Librariansh 2012;12(2):142-53. |
| 1 | Ellero NP. Crossing over: health sciences librarians contributing and collaborating on electronic medical record (EMR) implementation. J Hosp Librariansh 2009;9(1):89-107. |
| 1 | Epstein BA, Tannery NH, Wessel CB, Yarger F, LaDue J, Fiorillo AB. Development of a clinical information tool for the electronic medical record: a case study. J Med Libr Assoc 2010;98(3):223. |
| 1 | Fowler SA, Yaeger LH, Yu F, Doerhoff D, Schoening P, Kelly B. Electronic health record: integrating evidence-based information at the point of clinical decision making. J Med Libr Assoc 2014;102(1):52-5. |
| 1 | Friedman CP, Oxford GS, Juliano EL. A collaborative institutional model for integrating computer applications in the medical curriculum. Proc Annu Symp Comput Appl Med Care. 1991:752-6. |
| 1 | Fuller SS, Ketchell DS, Tarczy-Hornoch P, Masuda D. Integrating knowledge resources at the point of care: opportunities for librarians. Bull Med Libr Assoc 1999;87(4):393-403. |
| 2 | Geyer EM, Irish DE. Isolated to integrated: an evolving medical informatics curriculum. Med Ref Serv Q 2008;27(4):451-61. |
| 1 | Gomes AW, Linton A, Abate L. Strengthening our collaborations: building an electronic health record educational module. J Electron Resour Med Libr 2013:10(1):1-10. |
| 1 | Griffin-Sobel JP, Acee A, Sharoff L, Cobus-Kuo L, Woodstock-Wallace A, Dornbaum M. A transdisciplinary approach to faculty development in nursing education technology. Nurs Educ Perspect 2010;31(1):41-3. |
| 1 | Hannigan GG, Bartold SP, Browne BA, Fulton S, Henry BJ, Malcom P, et al. Computers and medical information: an elective for fourth-year medical students. Med Ref Serv Q 1996;15(4):81-88. |
| 1 | Hannigan GG, Edwards KA. Medical Informatics. Medical informatics in the medical school curriculum. Med Ref Serv Q 1996;15(1):71-76. |
| 1 | Harrod TP, Gomes AW. The experience of writing and teaching formative objective structured clinical examinations. Med Ref Serv Q 2017;36(2):111-119. |
| 1 | Hart JK, Newton BW, Boone SE. University of Arkansas for Medical Sciences electronic health record and medical informatics training for undergraduate health professionals. J Med Libr Assoc 2010;98(3):212. |
| 1 | Hersh W, Biagioli F, Scholl G, Gold J, Mohan V, Kassakian S, et al. From competencies to competence: model, approach, and lessons learned from implementing a clinical informatics curriculum for medical students. In: Shachak A, Borycki E, Reis SP, eds. Health professionals' education in the age of clinical information systems, mobile computing and social networks. St. Louis: Elsevier; 2017:269-87. |
| 1 | Hinegardner PG, Lansing PS. Nursing informatics programs at the University of Maryland at Baltimore. Bull Med Libr Assoc 1994;82(4):441-3 |
| 1 | King S, Murray H, MacDonald K. Evolving a collaborative matrix for teaching informatics online to health sciences students at the Massachusetts College of Pharmacy and Health Sciences. J Can Health Libr Assoc 2014;31(3):109-14. |
| 1 | Knehans A, Schirm V. Partnering to promote evidence-based practice by implementing Nursing Reference Center at the point of care. J Hosp Librariansh 2015;15(2):151-60. |
| 1 | Miles A. A solo hospital librarian's experience in clinical informatics. Med Ref Serv Q 2015;34(2):232-9. |
| 1 | Modica SF. The Camp PDA Experience. J Electron Resour Med Libr 2008;5(2):179-86. |
| 3 | Morgen EB. Implementing PDA technology in a medical library: experiences in a hospital library and an academic medical center library. Med Ref Serv Q 2003;22(1):11-9. |
| 1 | Newman M, Abernethy D, Wearne J. Why oh why HI? Using clinical audit to teach health informatics. 10th International Congress on Medical Librarianship. 2009. |
| 1 | Schulte SJ. Integrating information literacy into an online undergraduate nursing informatics course: the librarian's role in the design and teaching of the course. Med Ref Serv Q. 2008;27(2):158-172. |
| 1 | Schwartz B. The introduction of a medical informatics course into a medical school curriculum. Med Ref Serv Q 2011;30(1):74-82. |
| 1 | Stoddard MJ. Informatics Education. Handhelds in the health sciences library. Med Ref Serv Q 2001;20(3):75-82. |
| 1 | Tomasko JM, Adams NE, Garritano FG, Santos MC, Dillon PW. Collaborating to increase access to clinical and educational resources for surgery: a case study. J Surg Educ 2014;71(1):32-5. |
| 1 | Turman LU, Self PC, Calarco PV. Teaching a web‐based course in health informatics. Ref Serv Rev 2004;32(1):21-5. |
| 1 | Vuk J, Anders ME, Mercado CC, Kennedy RL, Casella J, Steelman SC. Impact of simulation training on self-efficacy of outpatient health care providers to use electronic health records. Int J Med Inform 2015;84(6):423-9. |
| 1 | Wallace RL. PDA training of faculty physicians. J Electron Resour Med Libr 2007;4(4):27-39. |
| 1 | Welton NJ. The University of Washington electronic medical record experience. J Med Libr Assoc 2010;98(3):217-9. |
| 1 | Yu X, Xie Y, Pan X, Mayfield-Johnson S, Whipple J, Azadbakht E. Developing an evidence-based public health informatics course. J Med Libr Assoc 2015;103(4):194-7. |
